# Post-Transplant Lymphoproliferative Disease (PTLD) after Allogeneic Hematopoietic Stem Cell Transplantation: Biology and Treatment Options

**DOI:** 10.3390/jcm11247542

**Published:** 2022-12-19

**Authors:** Michele Clerico, Irene Dogliotti, Andrea Aroldi, Chiara Consoli, Luisa Giaccone, Benedetto Bruno, Federica Cavallo

**Affiliations:** 1 Division of Hematology, Department of Molecular Biotechnology and Health Sciences, A.O.U. Città della Salute e della Scienza di Torino, University of Torino, 10126 Turin, Italy; 2 Stem Cell Transplant Unit, A.O.U. Città della Salute e della Scienza di Torino, 10126 Turin, Italy; 3 Division of Hematology, Bone Marrow Transplant Unit, University of Milan-Bicocca, Ospedale San Gerardo, 20900 Monza, Italy

**Keywords:** post-transplant lymphoproliferative disease (PTLD), allogeneic hematopoietic stem cell transplantation (alloHSCT), Epstein–Barr virus (EBV)

## Abstract

Post-transplant lymphoproliferative disease (PTLD) is a serious complication occurring as a consequence of immunosuppression in the setting of allogeneic hematopoietic stem cell transplantation (alloHSCT) or solid organ transplantation (SOT). The majority of PTLD arises from B-cells, and Epstein–Barr virus (EBV) infection is present in 60–80% of the cases, revealing the central role played by the latent infection in the pathogenesis of the disease. Therefore, EBV serological status is considered the most important risk factor associated with PTLDs, together with the depth of T-cell immunosuppression pre- and post-transplant. However, despite the advances in pathogenesis understanding and the introduction of novel treatment options, PTLD arising after alloHSCT remains a particularly challenging disease, and there is a need for consensus on how to treat rituximab-refractory cases. This review aims to explore the pathogenesis, risk factors, and treatment options of PTLD in the alloHSCT setting, finally focusing on adoptive immunotherapy options, namely EBV-specific cytotoxic T-lymphocytes (EBV-CTL) and chimeric antigen receptor T-cells (CAR T).

## 1. Introduction

Post-transplant lymphoproliferative disease (PTLD) is a rare and serious complication that occurs as a result of immunosuppression in the setting of allogeneic hematopoietic stem cell transplantation (alloHSCT) or solid organ transplantation (SOT) [1]. The estimated incidence varies according to the organ transplanted, about 2% in alloHSCT [2] and 10–15% in SOT [3].

Survival rates of PTLD have improved with the introduction of the anti-CD20 monoclonal antibody rituximab. However, reports on the potential cure of refractory PTLD are scarce. Today, there is no consensus on how to treat rituximab refractory PTLD, especially in highly aggressive diseases. In the present review, we will focus on PTLD following alloHSCT.

## 2. Pathophysiology of PTLD

The histological classification (World Health Organization, WHO, 2017) includes six types of morphological PTLD: plasmacytic hyperplasia, infectious mononucleosis-like, florid follicular hyperplasia, polymorphic, monomorphic (B-cell or T-/NK-cell types), and classical Hodgkin lymphoma PTLD. From a pathological point of view, monomorphic PTLD is not distinguishable from lymphomas with the same cell of origin in immunocompetent patients [4].

The majority of PTLD arises from B-cells; of note, latent Epstein–Barr virus (EBV) infection is present in 60–80% of the cases. This finding has led to intense research into the oncogenetic implications of EBV in this rare disease [5]. EBV-positive PTLD usually presents early in the post-transplant period, when the immunosuppression is deeper, while EBV-negative has a tendency to manifest later in the post-transplant period. The difference in presentation and treatment speaks of the difference in the pathophysiology of both entities [6].

Hodgkin lymphomas and PTLD of T-lineage (T-PTLD) are the rarest forms of monomorphic PTLD. Due to the rarity of T-PTLD, knowledge about pathogenesis, risk factors, therapy, and prognosis relies predominantly on case reports and small series. T-lymphocytes do not express the EBV receptor CD21; however, some T-PTLD might show aberrant T-cells, which are positive for both CD21 and EBV [7].

### 2.1. EBV-Positive PTLD

The pathophysiology of EBV-positive PTLD has been described. EBV virus is a latent gamma herpes virus with known oncogenic potential. Sero-epidemiology models suggest that 90% of the adult population has been infected, making it the most ubiquitous virus in humans [8]. Primary infection develops after contact with infected saliva or other secretions [9]. Although EBV is typical of recipient origin after SOT, donor-derived B-cells are usually the source of EBV-induced lymphoproliferation in HSCT [10].

During the acute phase of EBV infection, oral mucosa cells and B-cells become infected, leading to a robust immunogenic response with both antibody and cellular upregulation (via specific CD8 and CD4-positive T-cells) [11]; by mechanisms of immune tolerance and by establishing itself in memory B-cells, the EBV becomes latent and remains dormant in the reticuloendothelial system for the rest of the host life [1,12]. However, it may resurface in the event of profound immunosuppression, as in the post-transplant period [13].

**Figure 1:**
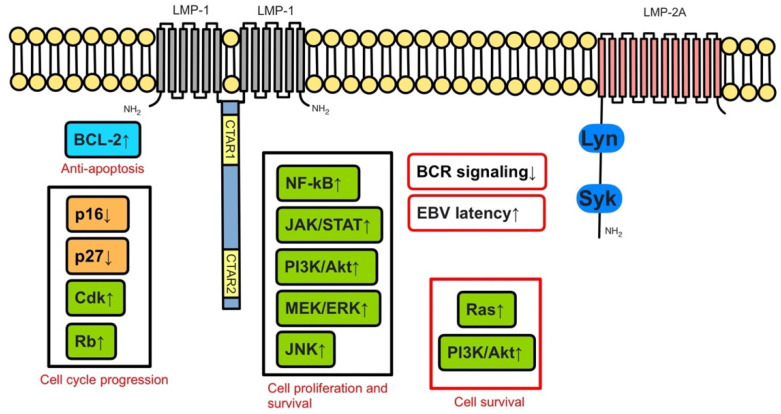
LMP-1, LMP-2A and downstream pathways in EBV-infected cells. Adapted from Ok, C., Li, L. & Young, K. EBV-driven B-cell lymphoproliferative disorders: from biology, classification and differential diagnosis to clinical management. *Exp Mol Med* 47, e132 (2015). https://doi.org/10.1038/emm.2014.82.

The oncogenic potential of EBV, especially in its latent phase, is demonstrated by the association with several malignancies, including endemic Burkitt lymphoma, Hodgkin lymphoma, and HIV-associated lymphomas other than PTLD [14]. Molecularly latent EBV infection is characterized by the expression of specific proteins by the infected cells, which include latent membrane protein 1 (LMP-1) and latent membrane protein 2A (LMP-2A). Among others, these proteins influence the cell cycle through several mechanisms, including a B-cell receptor-like signaling pathway [15,16] (Figure 1).

**Figure 2:**
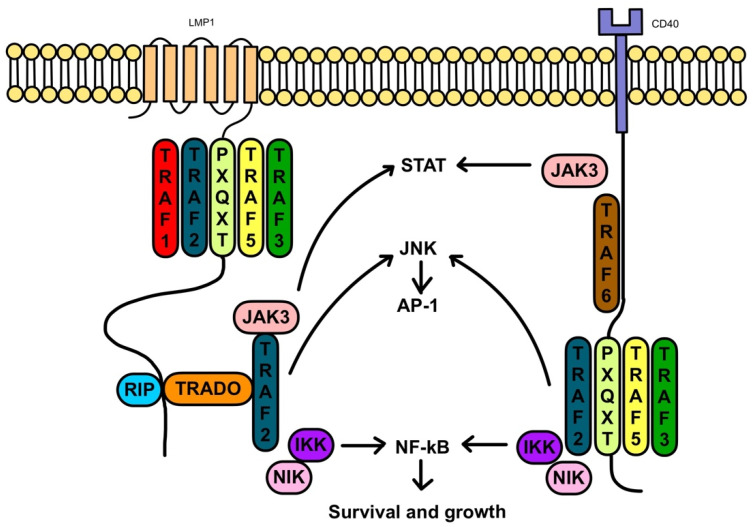
LMP-1 and signaling interactions with CD40. Adapted from Thorley-Lawson, D. Epstein-Barr virus: exploiting the immune system. *Nat Rev Immunol* 1, 75–82 (2001). https://doi.org/10.1038/35095584.

LMP-1 mimics CD40 and constitutively activates important proliferative signaling pathways in the B-cell cycle, such as NF-kB, JAK/STAT, ERK MAPK, IRF, and Wnt (Figure 2); it has been implicated in BCL-2 expression and blockage of apoptosis.

**Figure 3:**
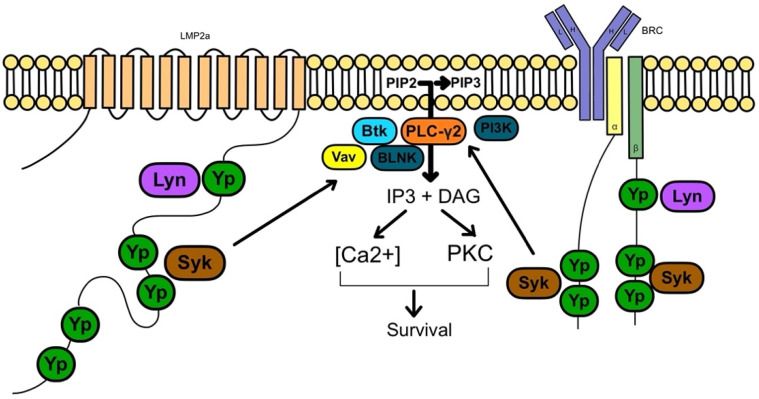
LMP-2A and signaling interactions with the B-cell receptor (BRC). Adapted from Thorley-Lawson, D. Epstein-Barr virus: exploiting the immune system. *Nat Rev Immunol* 1, 75–82 (2001). https://doi.org/10.1038/35095584.

LMP-2A interacts with the B-cell receptor signaling, and its pathway has been linked to anti-apoptosis (Figure 3). Ultimately, these molecular changes result in potential oncogenicity by leading to B-cell proliferation and transformation.

Under normal circumstances, the over-expression of LMP1 and LMP-2A by B-cells with latent EBV infection promotes a robust CD4 T-cell response that prevents the development of malignancies [17]. However, during post-transplant immunosuppression, the unchecked B-cell proliferation combined with the lack of T-cell activity promotes the development of PTLD. Mouse models show that the over-expression of LMP proteins combined with T-cell suppression results in fatal lymphoproliferative diseases [18].

### 2.2. EBV-Negative PTLD

EBV-negative PTLD is less common, often diagnosed later in the post-transplant period, and tends to have a poorer response to immunosuppression withdrawal [19]. The pathophysiology of EBV-negative PTLD is not well understood, though an increased expression of TP53 has been reported [20]. Genetic profiling has demonstrated important differences in the genomic landscape of EBV-positive and EBV-negative PTLD [21,22]. Some of the differences observed include more recurrent genomic lesions in EBV-negative PTLD, including the gain of 7p, del(4q25–q35), and gains of 7q, 11q24–q25 [23].

## 3. Risk Factors for PTLD

Two major features are related to the development of PTLD: (1) EBV serological status of transplant recipient and donor; and (2) depth of T-cell immunosuppression pre- and post-transplant. In particular, EBV serological status is considered the most important risk factor for post-SOT PTLDs (with relative risk, RR, up to 75) [24]: recipient-donor mismatch (R-/D+) determines a lack of a preformed anti-EBV cytotoxic immunity in the recipient. Therefore, the event of primary EBV infection in these patients triggers PTLD development. Since a negative EBV serological status at the time of transplant is typical of young age, pediatric patients show the highest risk [25].

For alloHSCT patients, ex vivo or in vivo T-lymphocyte depletion plays a major role. In fact, the use of anti-thymocyte globulin (ATG), commonly employed for matched unrelated donors, strongly affects PTLD development [26], similar to ex vivo T-cell depletion protocols, including the use of anti-CD52 alemtuzumab.

The role of other immunosuppressant drugs is more uncertain, partly due to different drug schedules and doses. High-intensity immunosuppression in the peri-transplant induction phase appears linked to early PTLDs, while the cumulative immunosuppressant burden in the following years affects late PTLDs [27]. Similarly, previous exposure to immunosuppressants as a treatment of the primary disease might also determine PTLD evolution [27]. Interestingly, a protective role of graft-versus-host disease (GvHD) prophylaxis with post-transplant cyclophosphamide (PTCY), commonly employed in haploidentical transplantation and, recently, also in matched-related and unrelated donors, has been suggested [22].

Recently, reduced intensity conditioning (RIC) regimens have been widely adopted to reduce transplant-related mortality and morbidity, allowing a widening of HSCT indications even to older patients or with a higher comorbidity index. The use of RIC regimens has been proven to be an independent risk factor for EBV-PTLD development, probably due to the more prolonged absence of normally restricting EBV-specific T-lymphocytes [28]. Similarly, it has been noted that previous splenectomy can independently increase PTLD risk; previous studies hypothesized that splenectomy might alter B-cells regulatory functions, thus leading to an increased EBV growth and PTLD occurrence [28].

Additional risk factors have been identified, such as the presence of the highly oncogenic variant protein LMP-1 in donors, an untreated HCV infection in recipients (that in turn might alter B-cell response and cause chronic antigenic stimulation), and non-Caucasian ethnicity [29,30]. Moreover, several reports have suggested that the reactivation of other viruses, such as CMV or BKV, probably via excessive immunosuppression, can trigger EBV reactivation, ultimately causing PTLD occurrence [27,31,32]. In particular, CMV reactivation has been linked with an increased EBV viral load: in one study, it was found to be the only statistically significant predictive factor for EBV reactivation in a multivariate analysis [31].

It has also been hypothesized that chronic antigen stimulation from viruses other than EBV might play a potential role in EBV-negative PTLDs [19]. In particular, HHV8 infection may underline effusive lymphoproliferative diseases.

PTLD occurrence after alloHSCT is primarily associated with the degree of HLA matching, given that more mismatches typically require stronger immunosuppression to prevent GvHD [27]. Thus, the presence of one HLA mismatch leads to an increased risk of PTLD (hazard ratio, HR, 5.8) as compared to HLA-identical sibling donors. Depending on the T-cell strategy, a haploidentical donor appears to increase the risk, although not quite as much as a matched unrelated donor (HR 3.9) [33]. No difference in PTLD incidence according to stem cell source (bone marrow vs. peripheral blood) has been reported, while an increased incidence has been associated with umbilical cord transplantation [34]. The presence of a pre-transplant indolent lymphoproliferative disorder does not increase PTLD risk [35].

Chronic GvHD is a potentially strong risk factor for late-onset PTLDs, both due to immune deregulation from GvHD itself and prolonged immunosuppression [33]. Little is known about the effect on PTLD development of novel targeted agents used in refractory GvHD, such as ruxolitinib, ibrutinib, or imatinib. Finally, age > 50 years represents an additional risk factor for alloHSCT recipients (RR for PTLD 5.1) [24].

In a review of 163 cases, alloHSCT was associated with early-onset T-PTLD, whereas late-onset occurred after immunosuppression with steroids and azathioprine without the administration of calcineurin inhibitors [36]. The major independent favorable prognostic factors were T-PTLD of the large granular lymphocytic leukemia subtype, young age, a combination of radiotherapy/radiochemotherapy, and reduced immunosuppression, whereas the hepatosplenic T-cell lymphoma subtype and cases with involvement of bone marrow, the central nervous system, or graft had an adverse prognosis [36].

## 4. Management

### 4.1. Reduction of Immunosuppression (RIS)

It is widely assumed that the reduction of the immunosuppression (RIS) is the typical first-line treatment for EBV-positive PTLD, though it may highly increase the risk of GvHD flare in alloHSCT and of rejection in SOT. Moreover, although data are scarce, RIS appears to have limited efficacy, and a specific treatment is necessary in almost the totality of cases.

### 4.2. Rituximab

The monoclonal anti-CD20 antibody rituximab is the gold standard of treatment in CD20+ PTLD, polymorphic PTLD, or monomorphic diffuse large B-cell lymphoma-like PTLD resistant to RIS [37,38]. However, a randomized phase II study has only been conducted in SOT [39]. Since most cases of PTLD in alloHSCT recipients are EBV related, the use of rituximab and its activity against EBV-infected B-lymphocytes (thus shifting the ratio in favor of antiviral/antitumor response driven by EBV-specific T-lymphocytes) has been studied in three settings: prophylaxis, preemptive therapy, and treatment of established CD20+ EBV-related PTLD.

#### 4.2.1. Prophylaxis

The administration of rituximab has been suggested in the prophylactic setting (before the onset of EBV DNAemia in seropositive patients) by a retrospective study of alemtuzumab-conditioned alloHSCT: an association between the use of rituximab within the 6 months prior to transplant and lack of EBV reactivation (EBV ≥ 500 copies/mL) was highlighted (HR 0.18, 95% CI 0.007–0.48, *p*-value 0.001), as well as its uses any time before transplant (HR 0.34, 95% CI 0.18–0.64, *p*-value 0.001) [40]. A recently published prospective study on cord transplant in patients conditioned with thymoglobulin compared the use of rituximab prior to transplant with no rituximab prophylaxis and demonstrated both a lower reactivation of EBV (13% vs. 2%) and a lower incidence of PTLD (8% vs. 0%) in the first group [41]. Finally, even though the use of rituximab might be followed by several possible side effects (i.e., prolonged hypogammaglobulinemia, neutropenia, and elevated risk of infections [42,43]), Besien et al. showed no differences in time to hematopoietic recovery, the incidence of CMV reactivation and of invasive infections [41].

#### 4.2.2. Preemptive Therapy

While the preemptive strategy is still debated in SOT [44], its role is particularly important in T-cell-depleted alloHSCT. Although there is no consensus on the EBV-DNA levels threshold for preemptive administration of rituximab, its use (375 mg/m^2^ once weekly until EBV-DNA negativity, usually 1–4 doses), combined with RIS whenever possible, is suggested by both American and European guidelines [45,46]. The threshold ranges from 1000 to 40.000 EBV copies/mL depending on the center-specific cut-off values and on the correlation between clinical and laboratory data [46]. A prospective study demonstrated significantly reduced rates of PTLD (18% vs. 49%) and completely abrogated PTLD mortality (0% vs. 26%, *p*-value = 0.04) with preemptive rituximab in patients receiving T-cell-depleted grafts [47]. Moreover, two large retrospective series showed high efficacy of preemptive rituximab, producing nearly 90% response rates and decreasing the incidence of PTLD [48,49]. More recent data suggest that a lower rituximab dose (100 mg/m^2^) may be equally effective [50].

#### 4.2.3. Treatment

As previously mentioned, there is a general paucity of solid data to guide the management of alloHSCT-associated PTLD [24]. However, European guidelines recommend the use of rituximab (375 mg/m^2^, once weekly for up to 4 doses) as first-line treatment for proven or probable EBV-PTLD combined with RIS, if clinically feasible [46]. In two retrospective studies, rituximab as frontline monotherapy reported response rates ranging from around 60% up to 84% when associated with RIS [51,52]. However, 2-year OS was only about 50% [51,52]. Rituximab may be associated with cellular immunotherapy, such as EBV-CTLs, to improve clinical outcomes [24,38].

### 4.3. Chemotherapy

The role of chemotherapy for EBV-PTLD after alloHSCT is limited to second-line therapies after rituximab failure and when novel cellular therapies are not readily available [46]. As a matter of fact, chemotherapy is poorly tolerated in alloHSCT patients with increased risks of prolonged neutropenia and graft failure [48]. A retrospective study clearly showed the limited efficacy of chemotherapy in patients who failed rituximab, where no complete remission was reported [38,53]. Despite these limitations, the most commonly used regimen is R-CHOP (rituximab, cyclophosphamide, hydroxydaunorubicin, doxorubicine, and prednisone) since its efficacy was shown in SOT [37].

### 4.4. Adoptive Immunotherapy

#### 4.4.1. EBV-Specific Cytotoxic T-Lymphocytes for PTLD

Immunotherapeutic approaches for the prevention and treatment of EBV-positive PTLD include the use of EBV-specific cytotoxic T-lymphocytes (EBV-CTL), which showed promising results in the setting of PTLD refractory to rituximab or other chemotherapy regimens [48]. It is important to point out that unselected donor lymphocytes infusions (DLI), which have initially been used to treat PTLD, showed clear efficacy with responses up to 70% though associated with a higher risk of GvHD that prevented their widespread feasibility in this setting [54]. By contrast, preselected donor EBV-CTL showed at least similar efficacy with a good toxicity profile without harboring an increased risk of GvHD [48,54].

EBV-CTL are CD3+ T-cells (both CD4+ and CD8+ subtypes at different ratios according to donor and selection technique) capable of recognizing EBV-associated antigens on tumor cells [55]. In one study, EBV-CTL showed promising activity both as a prophylactic and treatment strategy, preventing the onset of PTLD in a cohort of 101 high-risk HSCT patients and achieving a complete response in 11 out of 13 patients with documented PTLD [56]. Moreover, the efficacy of a pre-emptive strategy in eradicating EBV viremia had been previously reported with overall response rates (ORR) of up to 94% [48].

Although the potential benefits of EBV-CTL have clearly been validated, several hurdles have arisen and prevented their extensive clinical use. In fact, the manufacturing of this cell therapy turned out to be highly challenging, owing to the fact that donor EBV-CTL could not always be readily available or could be EBV negative as well. Thus, timely production may be an issue as PTLD can rapidly be progressive and difficult to contain [57].

In general, preselection of EBV-CTL was firstly performed by co-culturing donor-derived T-cells with EBV-infected lymphoblastoid cell lines (LCLs) in vitro, which provided selection and expansion of EBV-CTL from donor’s T-cell repertoire [48,57]. Unfortunately, the generation of donor EBV-CTL required several weeks, thus precluding the possibility of using this cell therapy immediately at the onset of PTLD [57,58,59]. Further strategies have been developed over the past decade [60,61]. Rather than the use of LCLs, one approach consisted of the rapid isolation of donor EBV-CTL by stimulating donor T-cells with a pool of EBV peptide epitopes from different EBV antigens, followed by isolation of active T-cells (expressing surface Interferon-γ [IFN-γ]) through antibody-mediated capture and immunomagnetic separation [60]. As a result, complete and prolonged remission was documented in patients at earlier stages of the disease, associated with a rapid and sustained reconstitution of a protective EBV-specific T-cell memory [60]. In addition, in case of poor manufacturing or in case of EBV-negative donors, “third-party” or “off-the-shelf” EBV-CTL have been considered. In a recent experience, EBV-CTL was obtained from HLA-matched and EBV-seropositive third-party blood donors. The selection was performed by matching EBV-CTL with the patient for at least 2 of 10 HLA alleles (HLA-A, -B, -C, -DR, or -DQ) and restricted by an HLA allele shared by EBV+ PTLD, transplant donor, and patient [61].

In this series, patients were treated within 1–2 days of referral, and ORR was 68% in 33 hematopoietic alloHSCT patients with rituximab refractory PTLD. Monomorphic diffuse large B-cell lymphoma [DLBCL] was the histological type in 80% of cases [61]. The maximal response was achieved after a median of two cycles, with 1-year survival in responding patients of 88.9% [61]. Of note, five patients with progressive PTLD received the second cycle of EBV-CTL from a different donor with the same HLA restriction, achieving an ORR in 60% of cases [61]. In the same study, another approach was the administration of EBV-CTL from a different donor with a different HLA restriction from the first donor. A durable response was obtained in four out of six patients with progressive PTLD [61].

Overall, several clinical trials are expanding the use of third-party EBV-CTL in the setting of PTLD. A phase III trial is exploring the administration of commercially available off-the-shelf, allogeneic EBV-CTL called Tabelecleucel for EBV+ PTLD patients who failed rituximab or rituximab and chemotherapy (ALLELE trial, NCT03394365). Preliminary results showed an ORR of 50% and a median duration of response not reached in responders [62]. Another phase I/II trial is currently evaluating the safety and efficacy of off-the-shelf EBV-peptide stimulated T-cells from matched or partially matched donors (EBV-TCL-01 trial, NCT02580539).

#### 4.4.2. Chimeric Antigen Receptor T-Cell Therapy for PTLD

Chimeric antigen receptor T-cell (CAR T-cell) therapy directed against CD19 surface antigen is a novel immunotherapeutic approach that demonstrated promising activity against relapsed and refractory DLBCL [63,64,65]. Either EBV-positive or EBV-negative DLBCL represents the vast majority of PTLD [37]. Though anti-CD19 CAR T-cell therapy might offer an opportunity for PTLD patients, sizable clinical trials evaluating the safety and efficacy of this novel cell therapy against PTLD are lacking, and, to date, only a few cases, primarily in the setting of SOT have been reported [66,67,68]. Unfortunately, the results were not satisfactory, and patients eventually progressed after transient response to CAR T-cells [66,67,68]. Continuous immunosuppression to avoid organ rejection and T-cell exhaustion have been considered factors that at least partly may explain the disappointing clinical outcomes with autologous anti-CD19 CAR T-cells [67]. In one report, autologous anti-CD19 CAR T-cells were combined with immune checkpoint blockade (anti-programmed cell death-1 [PD-1] antibody) in a PTLD patient with a prior allogeneic kidney transplant [69]. Unfortunately, partial remission was only transient. Moreover, the use of immune checkpoint inhibitors may as well lead to complete allograft rejection in SOT, which has not been described in this patient, probably due to continuous administration of tacrolimus [69,70].

To date, only one group recently reported successful treatment of PTLD after alloHSCT with donor-derived CAR T-cell therapy [71]. The treatment was based on the sequential infusion of anti-CD19 and anti-CD22 CAR T-cell products in order to improve T-cell performance by circumventing the risk of PTLD resistance through CD19-antigen loss, considered one of the most prevalent mechanisms of relapse after CAR T-cell infusion [72,73]. Of note, two patients with PTLD were successfully treated, achieving complete response with no recurrence of lymphadenopathy and persistent negative EBV viral load during follow-up [71]. Furthermore, the Authors suggested that manufacturing CAR T cells from donor-derived T-cells rather than from patient lymphoapheresis may have significantly helped to obtain higher performance CAR T. In fact, this would have helped obtain a high-performing T-lymphocytes product for CAR T-cell transduction, since an insufficient post-chemotherapy T-cell collection and/or T-cell dysfunction may heavily impair the final quality of CAR T-cell products [71].

Finally, another tumor-associated target is being explored for the treatment of EBV-positive cancers. Preclinical studies showed encouraging results using CAR T-cells directed against surface LMP1 expressed in EBV-positive cancers such as nasopharyngeal carcinoma [74]. An early phase I trial is now evaluating the use of anti-LMP1 CAR T-cell for the treatment of EBV/LMP1-positive cancers, including EBV-positive PTLD (NCT04657965). However, only future larger studies will determine the real role of adoptive cell therapies for the treatment of PTLD, particularly in the setting of alloHSCT.

## 5. Conclusions

New insights in the pathophysiology of PTLD and in the role of EBV infection have allowed remarkable advances in the treatment and in the improvement of clinical outcomes of this rare, but life-threatening complication of alloHSCT. EBV serological status and immunosuppressive strategies for transplantation remain the most relevant predictive factor for the onset of the disease. However the recent introduction of novel, effective adoptive immunotherapies represent an encouraging option for refractory cases.

## Data Availability

Not applicable.
